# Coupling enzymatic digestion with nanopore sensing for low-abundance EGFR-L858R mutation detection

**DOI:** 10.3389/fbioe.2025.1602494

**Published:** 2025-09-09

**Authors:** Ling Yan, Zhenxin Wang, Yajie Yin, Changchun Niu, Yang Luo

**Affiliations:** ^1^ Chongqing Medical University, Chongqing, China; ^2^ Chongqing Institute of Green and Intelligent Technology, Chinese Academy of Sciences, Chongqing, China; ^3^ Department of Laboratory Medicine, Chongqing General Hospital, Chongqing University, Chongqing, China; ^4^ Chongqing School, University of Chinese Academy of Sciences, Beijing, China

**Keywords:** single nucleotide variant, EGFR L858R, T7E1 digestion, nanopore detection, magnetic bead separation, tumor biomarker

## Abstract

Single nucleotide variants (SNVs) serve as crucial biomarkers for tumor diagnosis, treatment, and prognosis. However, their detection remains challenging due to the low mutation frequency and limited abundance. In this study, we developed a sensitive and efficient SNV detection method combining T7 endonuclease I (T7E1) digestion and nanopore detection. The assay was designed to detect the L858R mutation in exon 21 of the EGFR gene, a key driver mutation in various cancers. Magnetic bead probes were utilized to capture and purify target DNA, followed by enzymatic cleavage and nanopore detection. The optimal nanopore detection voltage was determined to be 500 mV, ensuring a clear and reproducible signal for identifying digested DNA fragments. This approach enabled the detection of L858R mutations with a sensitivity as low as 0.1%. Compared to conventional sequencing and digital PCR, our method is simple, time-efficient, and cost-effective. This strategy offers a promising platform for SNV detection, providing a valuable tool for tumor diagnosis and personalized treatment.

## 1 Introduction

Cancer is one of the leading causes of mortality worldwide (Sung et al., 2021), and its development is driven by a series of genetic mutations. Single nucleotide variants (SNVs), as the most common form of genetic mutations, have been widely recognized as key factors in cancer initiation, progression, and therapeutic response. In oncology, SNVs in key genes such as epidermal growth factor receptor (EGFR), tumor protein 53 (TP53), Kirsten rat sarcoma viral oncogene homolog (KRAS), breast cancer susceptibility gene 1 (BRCA1), BRCA2, and B-Raf proto-oncogene (BRAF), have been shown to influence tumor biology and therapeutic response. Approximately 10%–30% of patients with non-small cell lung cancer (NSCLC) harbor mutations in EGFR (Madeddu et al., 2022). The missense mutation EGFR L858R in exon 21, which accounts for 41% of all EGFR mutations, has been detected in lung cancer, colorectal cancer, and squamous cell carcinoma of the head and neck (Shigematsu and Gazdar, 2006). In addition, SNVs are closely associated with tumor drug resistance and prognosis (Gao et al., 2019; Yoon et al., (2024); Zeng et al., 2022). The detection of point mutations in genes is of great value in tumor screening, diagnosis, prognosis assessment, and precision treatment.

Over the past decade, advances in SNV detection technologies have revolutionized precision oncology by enabling high-resolution profiling of tumor-associated mutations. Traditional methods such as Sanger sequencing established the foundation for mutation analysis through chain-termination chemistry, offering high specificity but limited sensitivity, which restricts clinical utility in detecting low-abundance mutations (Joy et al., 2020). Next-generation sequencing (NGS) is a powerful, high-throughput method for detecting gene mutations, and it has become an important tool for cancer diagnosis and an integral part of patient management (Malapelle et al., 2023). NGS can process many samples simultaneously, enabling rapid and cost-effective SNV detection. It also allows the identification of previously unknown SNVs, which may lead to new discoveries and advances in genomic research. Although the cost of NGS has decreased over time, it is relatively expensive compared to other methods. Moreover, NGS generates vast amounts of data, requiring sophisticated bioinformatics tools and computational resources for data analysis and interpretation (Cabello-Aguilar et al., 2023; Daber et al., 2013). Digital PCR (dPCR) has recently emerged as a powerful method for SNV detection (Zhang et al., 2022). dPCR offers several advantages in SNV detection, and it provides increased sensitivity, allows the detection of rare SNVs present at low frequencies, and enables the absolute quantification of SNVs, providing accurate information on their abundance (Abi and Safavi, 2019). However, the cost of dPCR is relatively high because of the need for specialized equipment and reagents. Recently, numerous innovative methodologies have helped enhance sensitivity, specificity, and efficiency (Pinheiro et al., 2022; Tanaka et al., 2023). Emerging third-generation sequencing platforms like Oxford Nanopore and Pacific Biosciences offer unique advantages in detecting structural variations and phasing mutations through long-read sequencing (>10 kb reads) (Lee et al., 2021), but currently exhibit higher error rates compared to NGS (Logsdon et al., 2020). CRISPR/Cas12a technology combined with fluorescence or electrochemical detection can detect EGFR mutations at sub-femtomolar concentrations of up to 0.1% variant allele frequency (Lee et al., 2021). A direct mutation detection approach using a force-distance curve-based atomic force microscope can detect ctDNA with low mutant allele frequencies (0.006–0.2%) at high sensitivity and specificity (Mishra et al., 2021). However, these SNV detection methods have relatively low specificity and high false-positive probabilities. Recent advances in the detection of SNVs using nanotechnology have shown great promise (Mukhtar et al., 2021). Here, we report a novel SNV detection method based on solid nanopore technology with high selectivity, efficiency, and simplicity.

**SCHEME 1:**
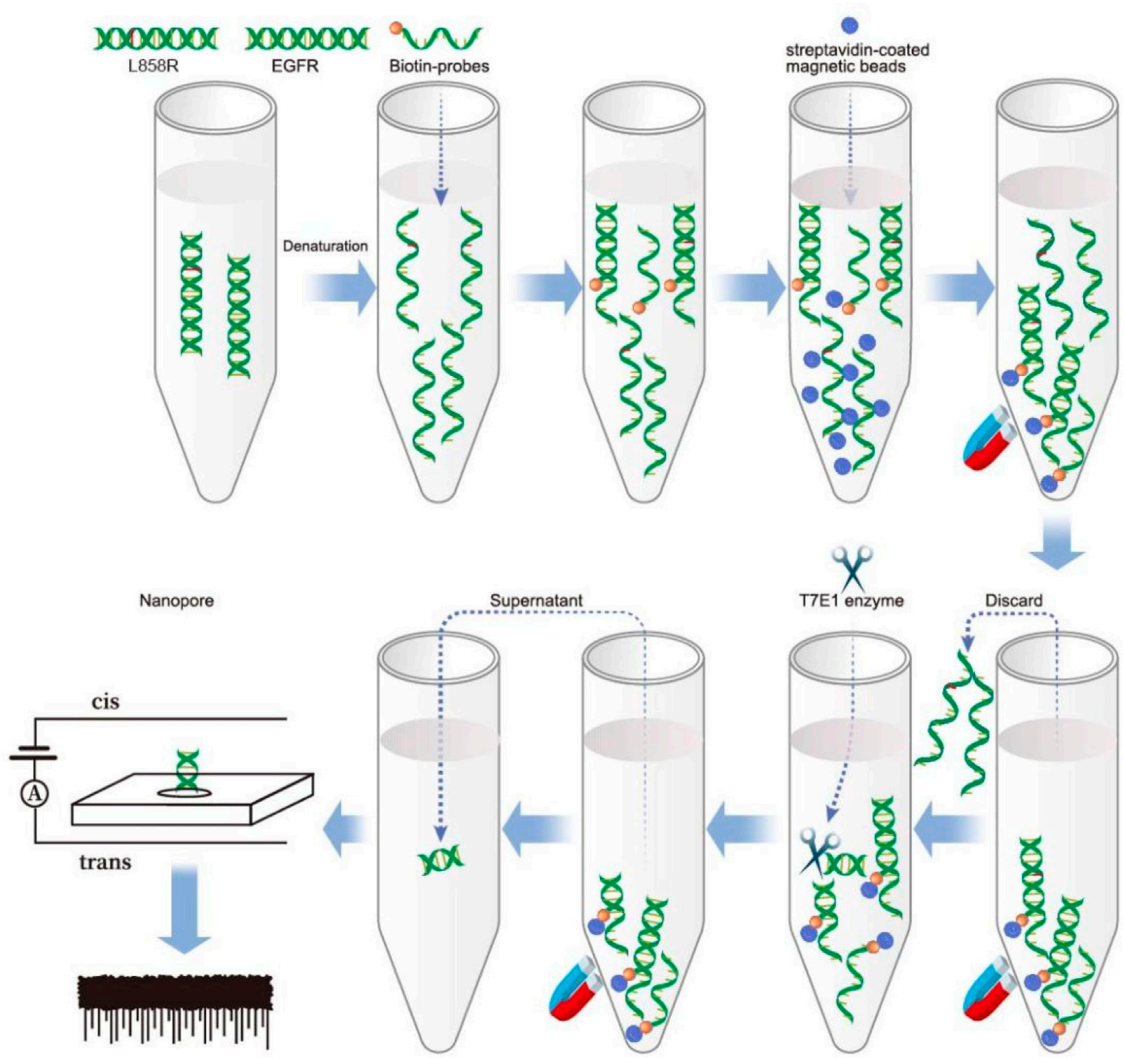
A schematic illustration of SNP detection using nanopore following T7E1 enzyme digestion.

Solid-state nanopores refer to nanoscale pores created in solid materials such as silicon or graphene. Compared with biological nanopores, solid nanopores have advantages such as tunability, stability, and ease of manufacturing (Yang et al., 2022). In nanopore devices, a nucleic acid sample is added to a reservoir and driven through a pore separated by a silicon nitride film sandwiched between two Ag/AgCl electrodes. When nucleic acid passes through a nanopore, the ion current is momentarily interrupted. At this point, a set of upward pulses appear in the current time trajectory, achieving the effect of detecting nucleic acids. Although the rapid translocation of DNA molecules through solid-state nanopores presents significant challenges for sequencing applications (Wei et al., 2022), substantial progress has been made in the analysis of nucleic acids in human plasma samples. Studies have shown that solid-state nanopore technology enables quantitative detection of specific nucleic acid sequences in human plasma, highlighting its broad potential for the analysis of viral, bacterial, and human genetic biomarkers (Elaguech et al., 2025; Alam et al., 2024). In this study, a biotin-labeled wild-type probe was hybridized with the target fragment, forming a heteroduplex with a single-base mismatch when the mutant sequence was present. The probe was then captured using streptavidin magnetic beads to purify the target fragments. The T7E1 enzyme was used to identify and cleave single-base mismatch sites (Guan et al., 2004; Vouillot et al., 2015). If the mutant gene is present, the supernatant obtained after magnetic separation contains cleaved nucleic acid fragments. Detection by solid-state nanopores generated upward pulses. The strategy adopted to detect SNV using solid-state nanopores is shown in Scheme 1.

## 2 Materials and methods

### 2.1 Heteroduplex formation

Oligonucleotides (Supplementary Table S1) were synthesized by Hippo Technologies Co. Ltd., China. For the formation of DNA segments of EGFR and L858R, the sense and antisense sequences were added to one tube, and for the formation of the heteroduplex of EGFR and L858R, the EGFR sense sequences and L858R antisense sequences were added to the tube and vortexed briefly. The mixture was denatured by heating at 95 °C for 5 min and then cooling down (−0.1 °C/3 s) to 25 °C using the Bio-Rad CFX96 Real-Time System (Bio-Rad, United States).

### 2.2 Capture the probe with streptavidin magnetic beads

A DNA capture probe (Supplementary Table S1) (Hippo Technologies Co. Ltd., China) was designed to hybridize with EGFR (fully complementary) and L858R (single-nucleotide mismatched), and it was modified with biotin at its 5′ends. Target DNA solutions were prepared in a 2× hybridization buffer (10× SSPE, 10× Denhardt’s, 10 mM EDTA, and 0.2% SDS). After heating to 95 °C for 5 min, the solution was mixed with the pre-warmed (2 min at 58 °C) capture probe and incubated at 58 °C for 4 h. The hybridization mix was then added to streptavidin magnetic beads (MedChemExpress). Streptavidin magnetic beads (Supplementary Figure S1) were prepared according to the manufacturer’s instructions. Briefly, 0.5 μL stock beads solution (10 mg/mL) was first washed three times with 200 μL wash buffer (1× TE, Yuanye Bio-Technology Co., Ltd. China; 1 M NaCl, GEN-VIEW Scientific, Inc. United States; 0.1% Tween-20, Sangon Biotech (Shanghai) Co., Ltd. China; pH 7.5), and the supernatant was removed after magnetic separation using a magnetic stand. Then, the magnetic beads were resuspended with a 20 μL wash buffer. The hybridization mix (20 μL) was added to the resuspended magnetic beads. After 1 h at 25 °C, the beads were removed and washed three times. The magnetic beads were then resuspended with 17 μL nuclease-free water.

### 2.3 Heteroduplex digestion by T7 endonuclease I (T7E1)

We added 1 μL T7E1 (New England Biolabs, United States) and 2 μL 1× NE buffer (50 mM NaCl, 10 mM Tris-HCl, 10 mM MgCl2, 1 mM DTT) to the 17 μL heteroduplexes. Then, we performed T7EI digestion at 37 °C using the Bio-Rad CFX96 Real-Time System.

### 2.4 Native polyacrylamide gel electrophoresis

Native polyacrylamide gel (12%) was prepared by mixing thoroughly 30% acrylamide-bis-acrylamide (29:1) 2.4 mL, 5× TBE buffer (89 mM boric acid, 2 mM EDTA, 89 mM Tris, pH 8.3) 1.0 mL, 60 μL of 10% ammonium persulfate, 6 μL tetramethyl ethylenediamine, and deionized water 2.4 mL. We loaded 5 μL DNA samples into the prepared native polyacrylamide gel, and the gel electrophoresis was performed in 1× TBE buffer at 150 V for approximately 1 h. The gels were imaged on a Chem-I Doc XRS system (Bio-Rad, United States) after staining with Realsafe-Red nucleotide gel stain (Real-Times Biotechnology Co., Ltd., China) for 30 min.

### 2.5 Fabrication of solid-state nanopore biosensor and data acquisition

Solid nanopores were prepared as described previously (Tian et al., 2023). Briefly, the chip (NORCADA Inc., Canada) was embedded in a customized flow cell filled with electrolytes of 10 mM Tris-HCl, 1 M KCl, and 1 mM ethylenediaminetetraacetic acid (EDTA, pH 8.0). Ag/AgCl was used as the reference electrode, and nanopores were prepared on the SiNx film using the electric pulse puncture method. Theoretical estimation of nanopore size can be made using the following formula: *G = σ(*4*L/π d*2*+*1*/d)-*1 (Yin et al., 2017; Kowalczyk et al., 2011). Where *G* is the conductance of the nanopore, which can be measured using I-V testing of the nanopore (Supplementary Figure S2), *σ* is the conductivity of the buffer solution (1 M KCl, 1 mM EDTA, 10 mM Tris, pH 7.6), σ at S·m^-1^, *L* is the length of the SiNx film (in this work, it is 20 nm), and *d* is the nanopore diameter. The fabricated nanopore chips were integrated into polydimethylsiloxane (PDMS) microfluidic chambers. Ag/AgCl electrodes were placed in each reservoir and connected to an Axopatch 700B pA current amplifier (Molecular Devices) to apply the transmembrane bias and record ionic currents through the nanopore. Between the analysis of two samples, the nanopore was rinsed three times with alcohol and deionized water to ensure cleanliness and prevent cross-contamination. Each sample was tested three times to ensure reproducibility.

### 2.6 Record and analysis of blockage currents produced by nucleotide

Nucleic acids were added to the *cis* side of the flow cell containing the solid-state nanopores. A bias voltage was applied to drive the nucleic acids through the pores for analysis. The current was measured using an Axopatch 200 B patch-clamp amplifier (Molecular Devices, CA, United States). The Clampfit and Origin software were used for signal extraction and data analysis, respectively.

### 2.7 Statistical analysis

All quantitative data are presented as means ± SD, statistical significance was evaluated using one-way ANOVA, followed by the Dunnett’s *t*-test.

## 3 Results and discussion

### 3.1 Formation and cleavage of DNA heteroduplexes with single-base mismatches and T7E1 digestion

**FIGURE 1:**
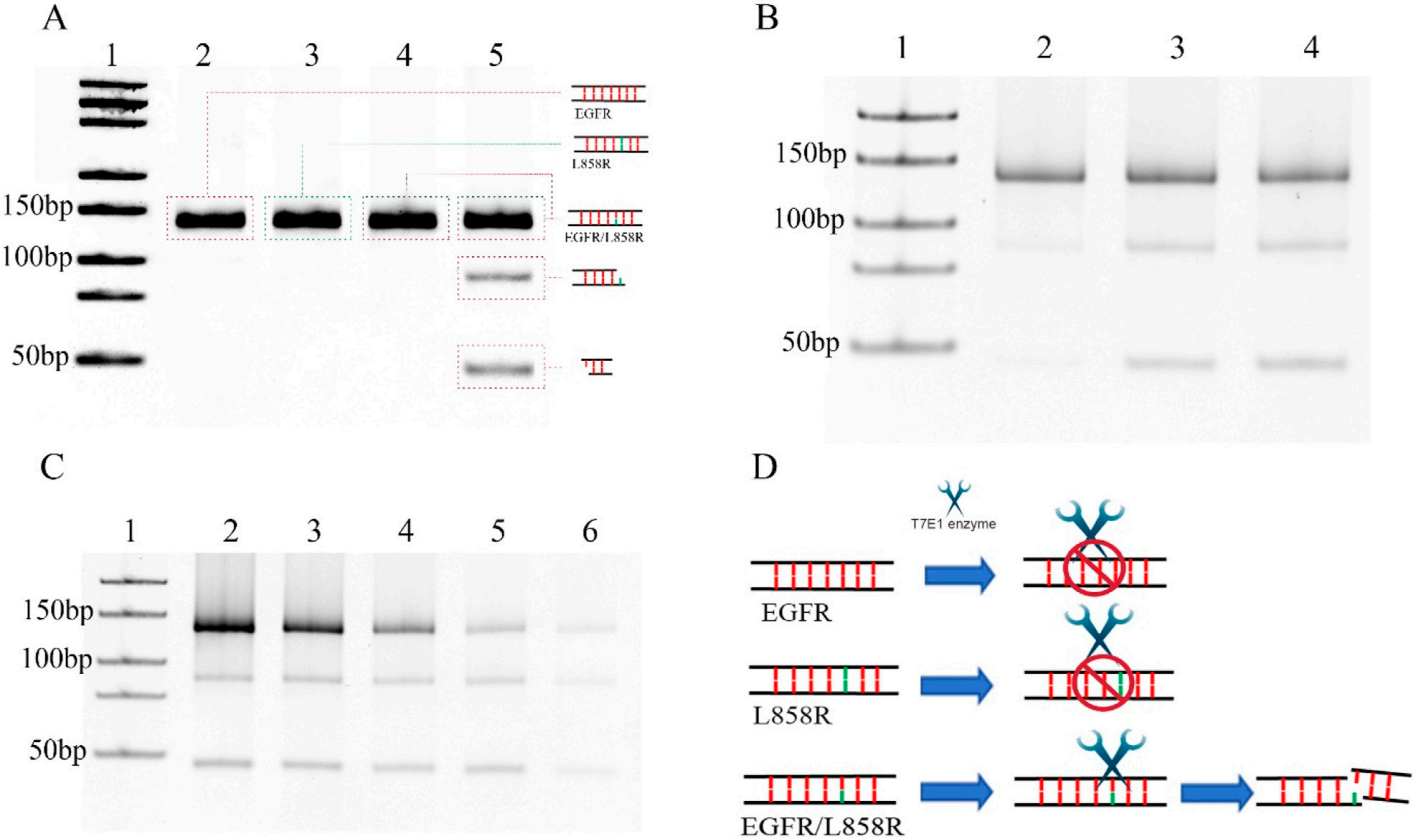
**(A)** Detection of DNA cleavage at single-base mismatches using the T7E1 enzyme. Lane 1: DNA marker (25-500bp), Lane 2: EGFR with digestion by T7E1 enzyme, Lane 3: L868R with digestion by T7E1 enzyme, Lane 4: EGFR/L858R without digestion, Lane 5: EGFR/L858R without digestion by T7E1 enzyme. **(B)** 0.3 μM EGFR/L858R heteroduplex digested at 37 °C using 5 units of T7E1 in a reaction volume of 20 μL for different times. Lane 1: DNA marker (25-500bp), Lane 2: Digested for 15 min, Lane 3: Digested for 30 min, Lane 4: Digested for 60 min. **(C)** Different concentration of EGFR/L858R heteroduplex digested at 37 °C using 5 units of T7E1 for 30 min in a reaction volume of 20 μL. Lane 1: DNA marker (25-500bp), Lane 2: 0.7 μM, Lane 3: 0.5 μM, Lane 4: 0.3 μM, Lane 5: 0.2 μM, Lane 6: 0.1 μM. **(D)** A schematic diagram of T7E1-mediated cleavage of the EGFR/L858R heteroduplex.

SNVs serve as critical predictors of tumor progression and therapeutic resistance while enabling the identification of actionable drug targets for personalized treatment strategies (Łaźniak et al., 2023; Watanabe et al., 2018; Kim et al., 2020; Alanee et al., 2017). However, detecting SNVs remains technically challenging due to their low mutation frequency (<5%) and single-base resolution requirements (Li et al., 2021). Despite these obstacles, advances in molecular technologies are gradually overcoming these difficulties, enabling the precise identification of oncogenic mutations that drive tumorigenesis. In this study, we synthesized DNA fragments containing mutations in the epidermal growth factor receptor (EGFR) gene to serve as detection targets (Supplementary Table S1). The EGFR L858R point mutation, located in exon 21 and classified as a type II kinase domain mutation, involves a thymine-to-guanine substitution at codon 858 of the EGFR gene. This nucleotide change leads to the replacement of leucine with arginine at the corresponding position in the EGFR protein (Liu et al., 2024). Positioned within the N-terminal region of the activation loop, the mutation replaces a hydrophobic leucine residue with a bulkier, positively charged arginine, which is structurally accommodated in the active conformation of the kinase (Yun et al., 2007). Functionally, L858R is an activating mutation that enhances EGFR’s catalytic efficiency by 20–50 times compared to the wild-type protein (Sutto and Gervasio, 2013). As a key oncogenic driver, L858R plays a critical role in the development of several malignancies, including lung, colorectal, and head and neck cancers (Andrews et al., 2023; Castellanos et al., 2017). The mutation detection strategy leverages two distinct biochemical properties: 1) the T7 endonuclease I (T7E1) enzyme’s ability to specifically recognize and cleave single-base mismatches, particularly those involving cytosine residues (Guan et al., 2004); and 2) nanopore-based single-molecule sensing capabilities. To investigate the enzymatic specificity, we systematically evaluated T7E1 activity against EGFR/L858R heteroduplex structures. T7E1 digestion was performed at its optimal temperature of 37 °C for 30 min (Supplementary Figure S3). Under these conditions, the enzyme specifically cleaved EGFR/L858R heteroduplexes at single-base mismatch sites, producing two distinct DNA fragments, whereas wild-type and mutant homoduplexes remained intact (Figure 1A). This finding confirms the enzyme’s discrimination capacity while revealing previously unreported cleavage dynamics specific to EGFR/L858R heteroduplexes. The experimental design addresses a critical knowledge gap, as the structural interplay between T7E1 enzymatic activity and EGFR mutation-bearing duplexes had not been systematically characterized prior to this investigation.

### 3.2 Optimization of parameters of T7E1 digestion

T7E1 is a key enzyme used in genetic engineering and molecular biology, and it is a type of endonuclease that is commonly used for gene editing and mapping. Moreover, it can be used for single nucleotide polymorphism (SNP) detection owing to its ability to recognize and cleave DNA strands containing mismatched base pairs. The T7E1 enzyme is often employed in conjunction with other molecular tools, such as CRISPR/Cas9, to induce double-stranded DNA breaks at mismatched sites in the genome. The optimal temperature for the T7E1 digestion is 37 °C. In previous experiments, five to ten units of the T7E1 enzyme could achieve high cleavage efficiency for 100 ng of DNA in a 20 μL reaction system (Zou et al., 2023; Seol et al., 2024). In this experiment, we first performed T7EI digestion at 37 °C for 30 min using five units of T7E1 in a reaction volume of 20 μL (Figure 1A). The optimal enzymatic efficiency was achieved when the enzyme time was 30 min, whereas increasing the time to 1 h did not significantly improve the enzymatic efficiency of the T7E1 enzyme (Figure 1B). Increasing the enzyme concentration or cutting time may lead to the nonspecific cutting of fragments. The DNA concentration was then changed. The results indicate that although the amount of cleavage products decreased with lower DNA concentrations, more than half of the mismatched DNA at a concentration of 0.1 μM was still digested by five units of T7E1 at 37 °C for 30 min in a 20 μL reaction volume (Figure 1C). This suggests that T7E1 is sufficiently efficient for detecting low concentrations of EGFR/L858R heteroduplexes under these conditions (Figure 1D). Thus, using five units of T7E1 at 37 °C for 30 min provides adequate enzymatic efficiency for subsequent analysis.

### 3.3 Target DNA capture and separation

**FIGURE 2:**
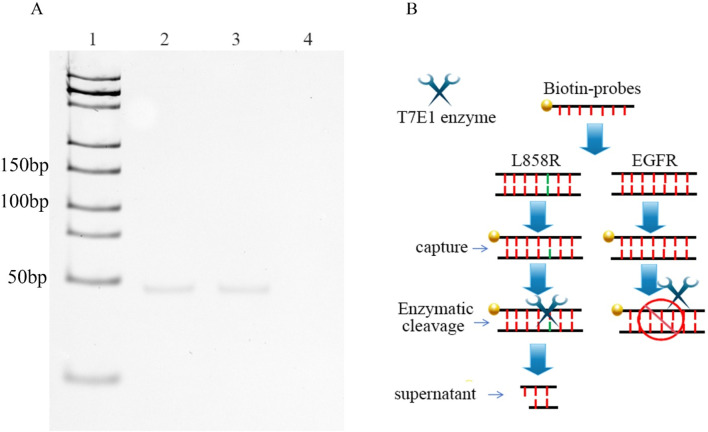
**(A)** DNAs were captured by probes, and then digested by T7E1. The DNA fragments, generated after digestion, were finally purified using magnetic beads. Lane 1: DNA marker (25-500bp); Lane 2: 0.3 μM EGFR/L858R heteroduplex; Lane 3: 0.3 μM L858R, Lane 4: 0.3 μM EGFR. **(B)** Workflow for nanopore detection of enzyme-digested nucleic acid fragments.

Magnetic separation is an efficient technology for separating and purifying nucleic acids from chemical and biological suspensions (Berensmeier, 2006). For target DNA capture, a DNA capture probe modified with 5′-biotin was designed and custom-synthesized. The probe was fully complementary to EGFR but mismatched with L858R. To enhance binding specificity and stabilize interaction with a target containing a single-base mismatch, we designed a 130-bp probe (Supplementary Table S1). (Supplementary Table S1). Excess probes were added to mixtures containing the wild-type and mutant genes. After denaturation and gradual annealing, the probes hybridized randomly with the wild-type and mutant DNA. Streptavidin magnetic beads were used to separate the probes. Nucleic acids that were not bound to the probes were discarded along with the supernatant. If the sample contains a mutation, the probes can capture it and form single-base-mismatch heteroduplexes. Thus, a single-base mismatch could be recognized and cleaved by the T7E1 enzymes. The oligonucleotides combined with the magnetic beads through the probes were removed using magnetic separation, and the free fragment nucleotides were left in the supernatant. In this study, our results showed that 46 bp fragment nucleotides were detected by native polyacrylamide gel electrophoresis in the L858R-containing mixture after T7E1 enzyme digestion and magnetic separation (Figure 2). No band was detected for EGFR without L858R (Figure 2). In conventional detection, interference may arise not only from wild-type EGFR but also from other normal or mutated genes in the human genome. To further verify the specificity of our method, we conducted validation experiments using the K-RAS gene and its G12D mutant, which are unrelated to EGFR but commonly found in cancer samples. Results showed that no bands were observed for the K-RAS gene or samples containing the G12D mutation, indicating that the capture probe is specific to EGFR and cannot capture K-RAS or G12D (Supplementary Figure S4). However, native polyacrylamide gel electrophoresis cannot detect low concentrations of nucleotides. Therefore, we developed a highly sensitive nanopore detection method.

### 3.4 Exploration of optimal nanopore detection voltage

**FIGURE 3:**
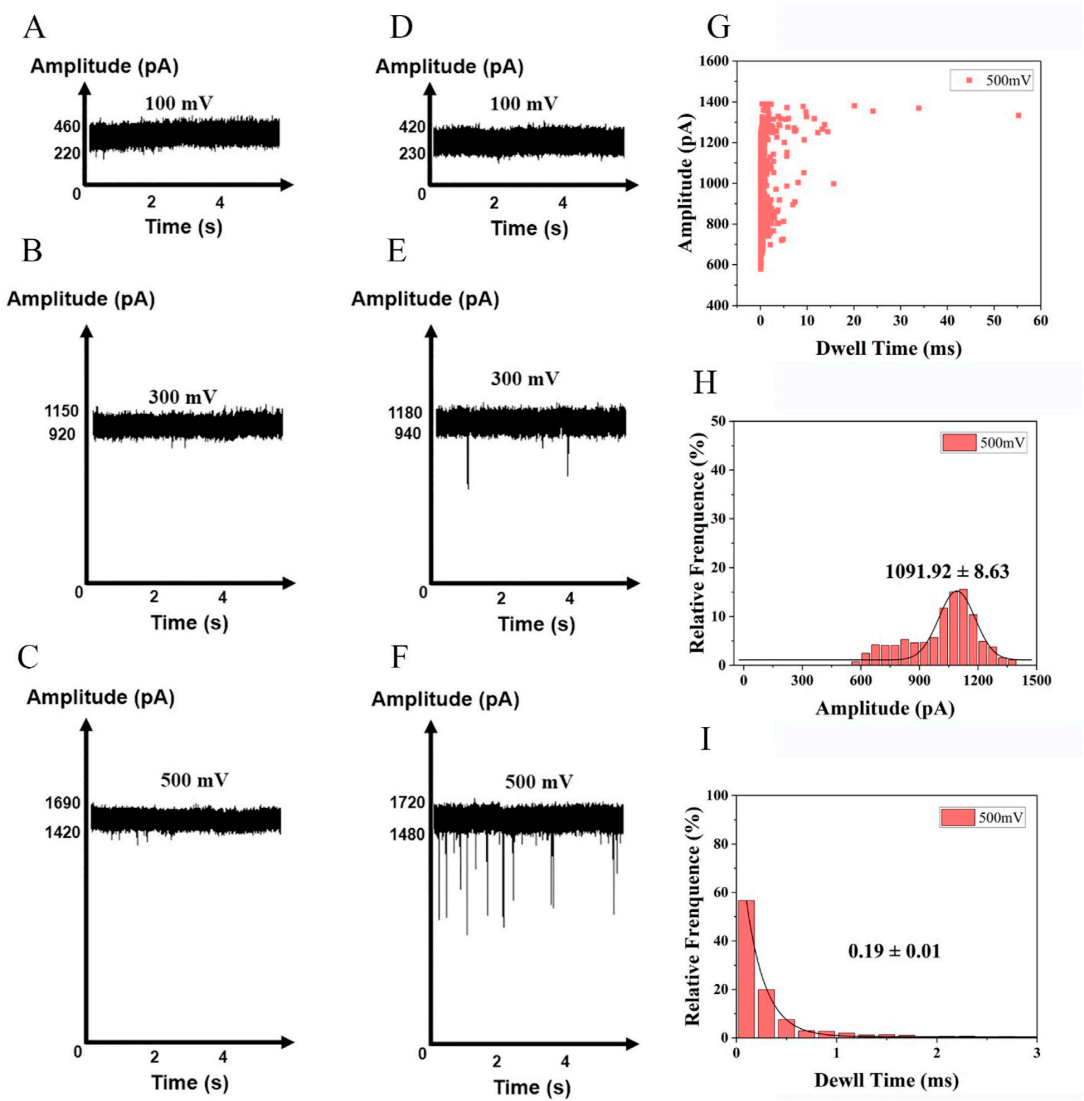
Detection of nucleotides at a series of voltages +100 mV, +300 mV and +500 mV in 1 M KCl,10 mM Tris, 1 mM EDTA, pH 8.0. **(A)** buffer at +100 mV, **(B)** buffer at +300 mV, **(C)** buffer at +500 mV, **(D)** nucleotides at+100 mV **(E)** nucleotides at +300 mV **(F)** nucleotides at +500 mV (Events: 329 ± 28/min). Synthesized nucleic acid fragment detected by nanopore at +500 mV: **(G)** Scatter plots of the amplitude of current blockade with dwell time; **(H)** Gaussian distributions of normalized histograms for current amplitude; **(I)** Normalized histograms of dwell time with fitting method of exponential decay.

To determine a suitable nanopore detection voltage, we synthesized a nucleic acid fragment with the same sequence as the free fragment (Supplementary Table S1) after enzyme digestion. The translocation of the DNA fragments into the nanopore was investigated at a series of voltages ranging from +100 to +500 mV. The nanopores employed in our experiments had an approximate diameter of 3 nm. In previous studies, the 3 nm nanopore demonstrated superior analytical performance for nucleic acid analysis (Alam et al., 2024). DNA fragments at a final concentration of 10 nM were added to the *cis* chamber of the flow cell. The translocation of molecules through nanopores requires overcoming free energy barriers, such as geometric confinement at the pore entrance and electrostatic repulsion (Chinappi et al., 2020). At low voltages, the electrophoretic force is insufficient to overcome these barriers, preventing effective entry into the nanopore. When voltages of +100 and +300 mV were applied, no identifiable signals were observed under voltage-clamp conditions. Therefore, there was no blocking signal in the current trajectory and no discernible signals were detected in DNA-free blank samples (Figure 3A-E). Consequently, a threshold voltage (500 mV) is necessary to lower the barrier *via* enhanced electric field energy, leading to a nonlinear relationship between the capture rate and the applied voltage (Grosberg and Rabin, 2010). The observed open-pore current was around 1,425 pA. Thus, a series of current signals was detected at 500 mV (Figure 3). The raw translocation traces are shown in Figure 3F, and the scatter plots are presented in Figure 3G. According to the Gaussian fit of the histogram, the average blocking amplitude at 500 mV was 1,091.92 ± 8.63 pA (Figure 3H). Under an applied voltage of 500 mV, the average residence time of the blocking signal was 0.19 ± 0.01 m (Figure 3I).

### 3.5 Detection of target L858R gene

**FIGURE 4:**
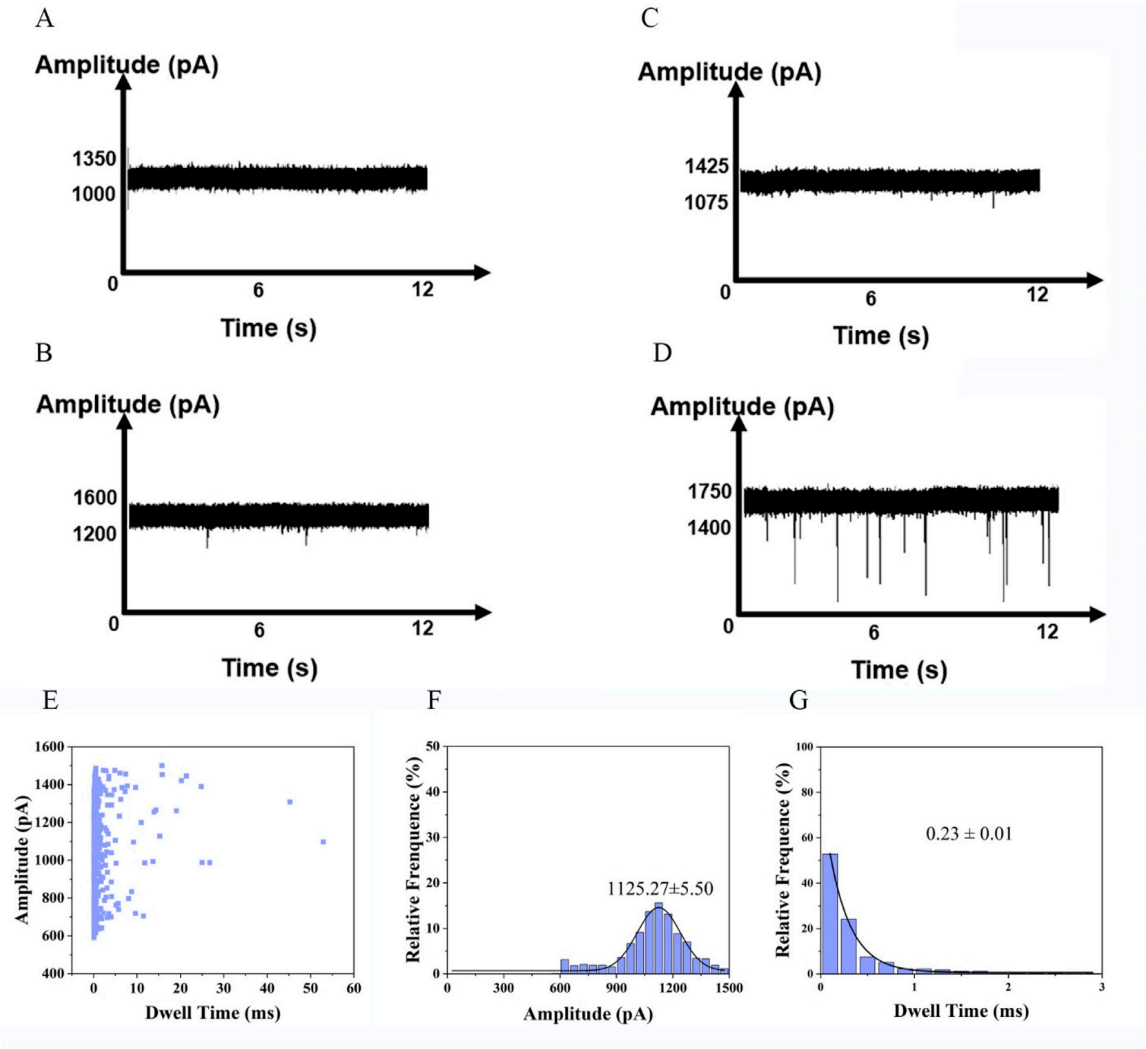
DNA fragments generated after T7E1 digestion detected by nanopore. **(A)** Current signal in the presence of buffer. **(B)** Current signal in the presence of T7E1 enzyme. **(C)** Current signal in the presence of EGFR wild type. **(D)** Current signal in the presence of L858R (Events: 54 ± 7/min). **(E)** Scatter plots of the amplitude of current blockade with dwell time; **(F)** Gaussian distributions of normalized histograms for current amplitude; **(G)** Normalized histograms of dwell time with fitting method of exponential decay.

To evaluate the feasibility of nanopore detection of enzyme digestion products, we measured translocation events at a voltage of 500 mV. As negative controls, samples containing only buffer (Figure 4A) or enzyme (Figure 4B) showed no detectable pulses. Similarly, no signals were observed for wild-type EGFR (Figure 4C), as the absence of mismatches prevented enzymatic digestion. In samples containing the L858R-mutated EGFR gene, hybridization with the probe introduced a single-base mismatch, which enabled T7E1 to recognize and cleave the heteroduplex, releasing short nucleic acid fragments. After magnetic bead separation, free nucleic acids in the supernatant generated discernible pulse signals in nanopore detection (Figure 4D). The raw translocation traces and scatter plots are shown in Figure 4E. The amplitude of the current blockade was 1,125.27 ± 5.50 pA (Figure 4F). Histograms of the dwell times are shown in Figure 4G. Specificity was further validated using the K-RAS gene and its G12D mutant; in this case, probe incompatibility resulted in no digestion or nanopore signals (Supplementary Figure S5). These results confirm the system’s ability to discriminate single-nucleotide variations with minimal background interference, underscoring its potential for precise mutation detection.

### 3.6 Low-abundance assay of SNV targets

**FIGURE 5:**
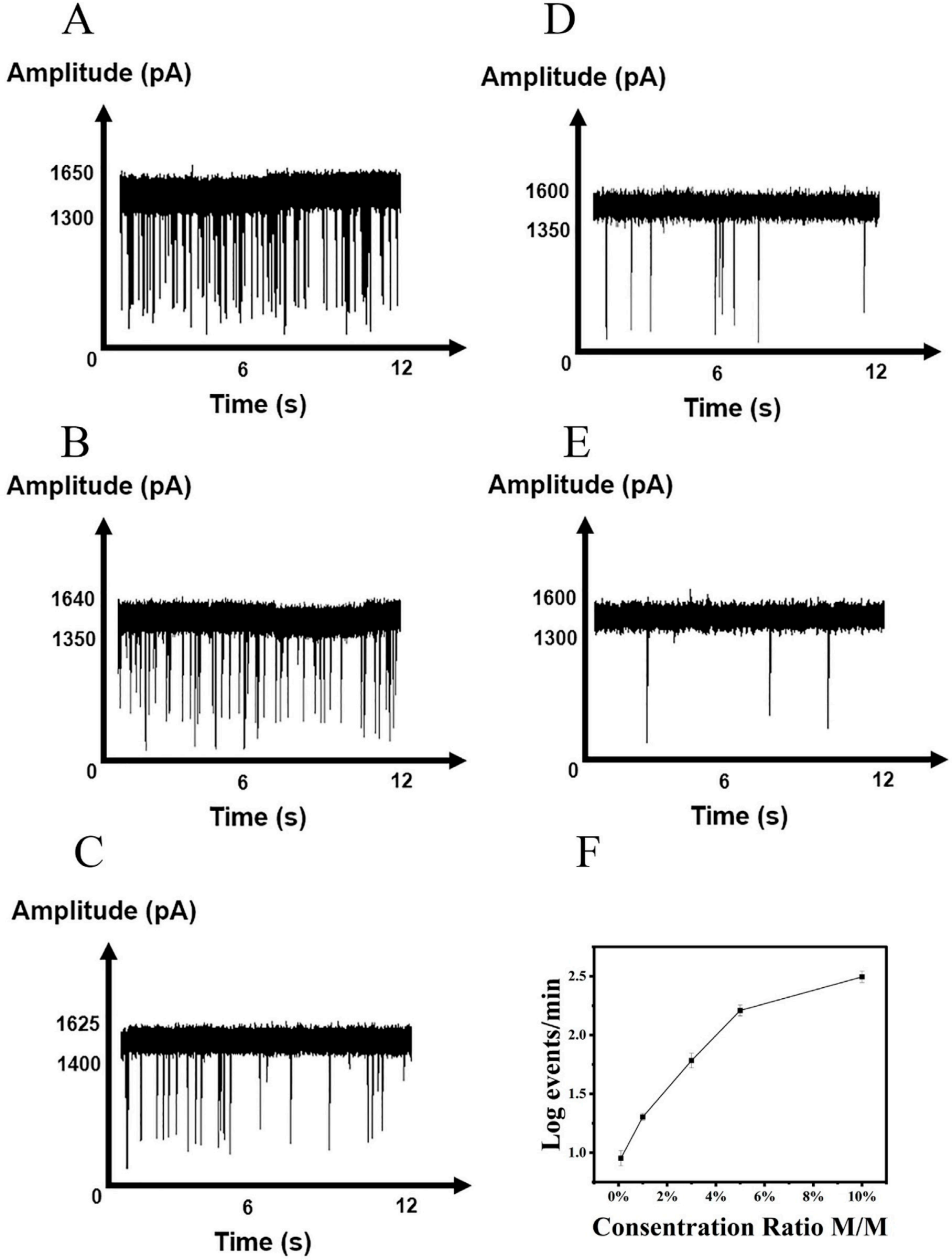
Detection of L858R at various concentration ratio. **(A)** 10%, **(B)** 5%, **(C)** 3%, **(D)** 1%, **(E)** 0.1%. **(F)** Correlations of the event frequency with the concentrations of L858R.

Detecting SNVs presents several challenges, primarily due to the need for high sensitivity and specificity. The low abundance of certain variants, particularly in heterogeneous populations, makes it difficult to distinguish them from background noise. In our study, 50 μL of synthesized EGFR and L858R gene solutions (0.1 nM) were aliquoted into a series of centrifuge tubes, with L858R-to-EGFR concentration ratios set at 10%, 5%, 1%, 0.3%, and 0.1%. After magnetic bead enrichment and purification, enzyme digestion, and repurification, mutated genes were detected at levels as low as 0.1% (Figure 5). The raw translocation traces and scatter plots are shown in Supplementary Figure S6A–E. The amplitudes of the current blockade are shown in Supplementary Figure S6F–J. Supplementary Figure S6H–O shows the histograms of dwell times. This method demonstrates effective SNV detection, with a detection limit of 0.1%, compared with 0.5% for NGS (Yang et al., 2017). Although a positive correlation between nanopore signal intensity and variant allele frequency (VAF) was observed, under the current experimental conditions, the reproducibility and linearity of this correlation were limited, preventing the establishment of a fully reliable calibration curve or regression model. Therefore, the assay is currently presented as a qualitative method for detecting the target mutation. Further optimization may enhance its potential for quantitative applications in future studies.

## 4 Conclusion

SNVs are common variations in human genes and are promising biomarkers for clinical and biological applications. SNV detection plays a critical role in tumor diagnosis, treatment, and prognosis (Cui et al., 2023; Jacob et al., 2020). In this study, a magnetic bead probe was employed to enrich and purify the target gene. A sensitive SNV detection method was then developed by combining the specific recognition and endonucleolytic cleavage activity of the T7E1 enzyme for base mismatches with a nanopore detection platform. Compared to second-generation sequencing technology and digital PCR, this method is relatively simple, time-saving, and inexpensive. In addition to the EGFR L858R mutation, the proposed method is potentially applicable to the detection of other clinically relevant SNVs. The T7E1 enzyme recognizes and cleaves heteroduplex DNA formed between mutant and wild-type sequences based on the presence of base mismatches, regardless of the specific codon involved. Therefore, as long as an appropriately designed probe forms a mismatch-containing heteroduplex with the target sequence, the corresponding SNV can, in principle, be detected using this approach.

## Data Availability

The original contributions presented in the study are included in the article/Supplementary Material, further inquiries can be directed to the corresponding authors.
