# Supplementary material for: Coupling enzymatic digestion with nanopore sensing for low-abundance EGFR-L858R mutation detection
Source: Front Bioeng Biotechnol. 2025 Sep 9;13:1602494. doi: 10.3389/fbioe.2025.1602494 (PMC12454318; doi:10.3389/fbioe.2025.1602494)
Supplement: Supplementary file 1 [file Supplementaryfile1.docx]

SUPPORTING INFORMATION

Coupling Enzymatic Digestion with Nanopore Sensing for Low-Abundance EGFR-L858R Mutation Detection

Ling Yan^abc^, Zhenxin Wang^bd^, Yajie Yin^bd^, Changchun Niu^abc^*, Yang Luo^ac^*

a Chongqing Medical University, Chongqing, 400016, China

b Chongqing Institute of Green and Intelligent Technology, Chinese Academy of Sciences, Chongqing, 400714, China

c Department of Laboratory Medicine, Chongqing General Hospital, Chongqing University, Chongqing, 401147, China

d Chongqing School, University of Chinese Academy of Sciences, Beijing, 400714, China

*Corresponding author:

Chongqing Medical University, Chongqing, 400016, China

Yang Luo, [luoy@cqu.edu.cn](mailto:luoy@cqu.edu.cn); Changchun Niu, 52736601@qq.com.Table S1: The oligonucleotides used in the experiments

| Name | Sequence |
| --- | --- |
| EGFR-sense | 5’-CCG TCG CTT GGT GCA CCG CGA CCT GGC AGC CAG GAA CGT ACT GGT GAA AAC ACC GCA GCA TGT CAA GAT CAC AGA TTT TGG GCT GGC CAA ACT GCT GGG TGC GGA AGA GAA AGA ATA CCA TGC AGA AGG A-3’ |
| EGFR-antisense | 5’-TCC TTC TGC ATG GTA TTC TTT CTC TTC CGC ACC CAG CAG TTT GGC CAG CCC AAA ATC TGT GAT CTT GAC ATG CTG CGG TGT TTT CAC CAG TAC GTT CCT GGC TGC CAG GTC GCG GTG CAC CAA GCG ACG G-3’ |
| L858R-sense | 5’-CCG TCG CTT GGT GCA CCG CGA CCT GGC AGC CAG GAA CGT ACT GGT GAA AAC ACC GCA GCA TGT CAA GAT CAC AGA TTT TGG GCG GGC CAA ACT GCT GGG TGC GGA AGA GAA AGA ATA CCA TGC AGA AGG A-3’ |
| L858R- antisense | 5’-TCC TTC TGC ATG GTA TTC TTT CTC TTC CGC ACC CAG CAG TTT GGC CCG CCC AAA ATC TGT GAT CTT GAC ATG CTG CGG TGT TTT CAC CAG TAC GTT CCT GGC TGC CAG GTC GCG GTG CAC CAA GCG ACG G-3’ |
| K-Ras -sense | 5’-TTC TAA TAT AGT CAC ATT TTC ATT ATT TTT ATT ATA AGG CCT GCT GAA AAT GAC TGA ATA TAA ACT TGA GGT AGT TGG AGC TGG TGG CGT AGG CAA GAG TGC CTT GAG GAT ACA GCT AAT TCA GAA TCA T-3’ |
| K-Ras -antisense | 5’-ATG ATT CTG AAT TAG CTG TAT CGT CAA GGC ACT CTT GCC TAC GCC ACC AGC TCC AAC TAC CAC AAG TTT ATA TTC AGT CAT TTT CAG CAG GCC TTA TAA TAA AAA TAA TGA AAA TGT GAC TAT ATT AGA A-3’ |
| G12D -sense | 5’-TTC TAA TAT AGT CAC ATT TTC ATT ATT TTT ATT ATA AGG CCT GCT GAA AAT GAC TGA ATA TAA ACT TGA GGT AGT TGG AGC TGT TGG CGT AGG CAA GAG TGC CTT GAG GAT ACA GCT AAT TCA GAA TCA T-3’ |
| G12D -antisense | 5’-ATG ATT CTG AAT TAG CTG TAT CGT CAA GGC ACT CTT GCC TAC GCC AAC AGC TCC AAC TAC CAC AAG TTT ATA TTC AGT CAT TTT CAG CAG GCC TTA TAA TAA AAA TAA TGA AAA TGT GAC TAT ATT AGA A-3’ |
| DNA capture probe | 5’-CCG TCG CTT GGT GCA CCG CGA CCT GGC AGC CAG GAA CGT ACT GGT GAA AAC ACC GCA GCA TGT CAA GAT CAC AGA TTT TGG GCT GGC CAA ACT GCT GGG TGC GGA AGA GAA AGA ATA CCA TGC AGA AGG A-3’ |
| Digested Fragment | 5’-GGC CAA ACT GCT GGG TGC GGA AGA GAA AGA ATA CCA TGC AGA AGG A-3’ |


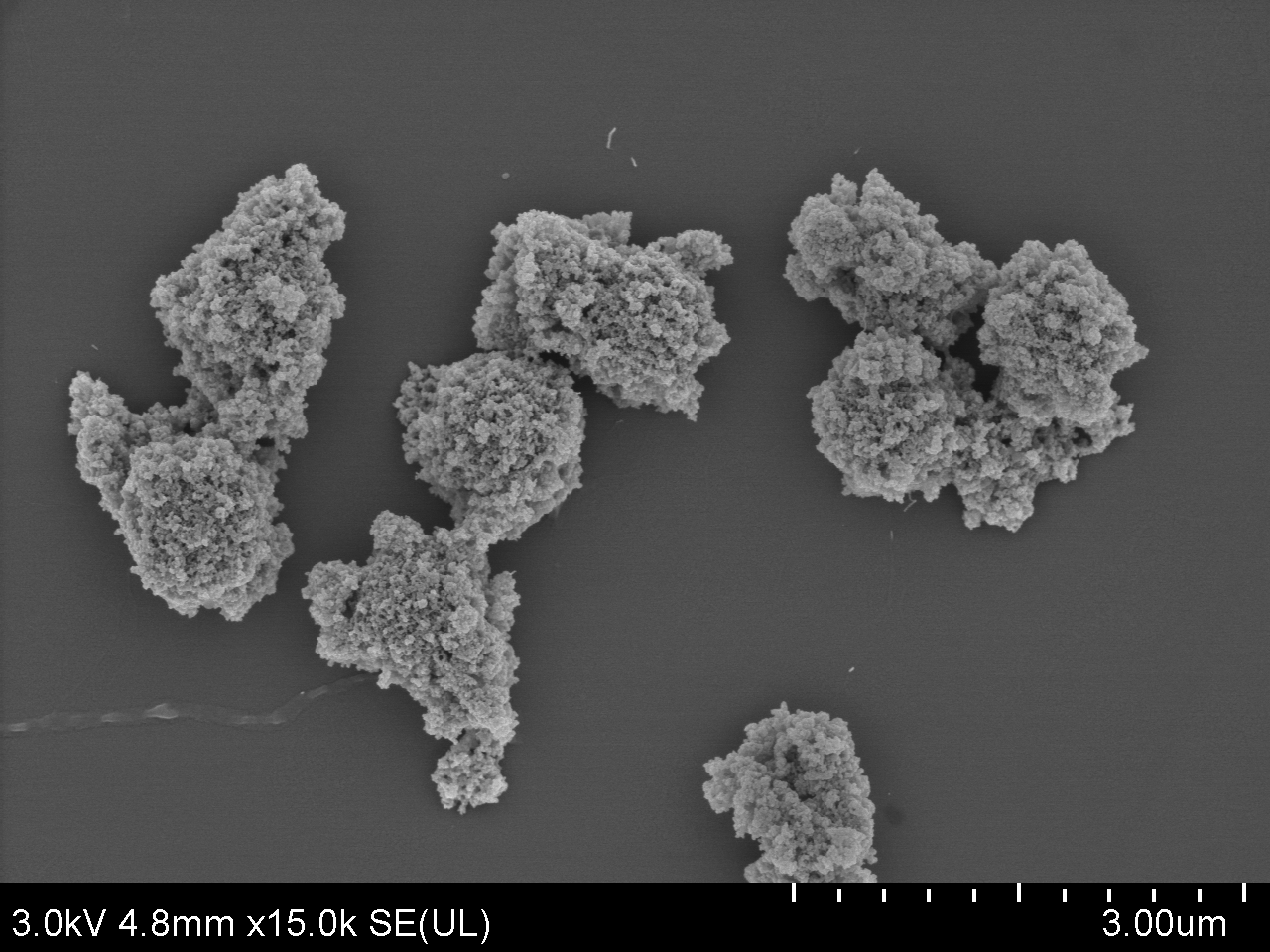


Fig S1 Scanning electron microscopy (SEM) image of 1 µm streptavidin-coated magnetic beads, showing a spherical morphology with surface roughness and thickening characteristics.


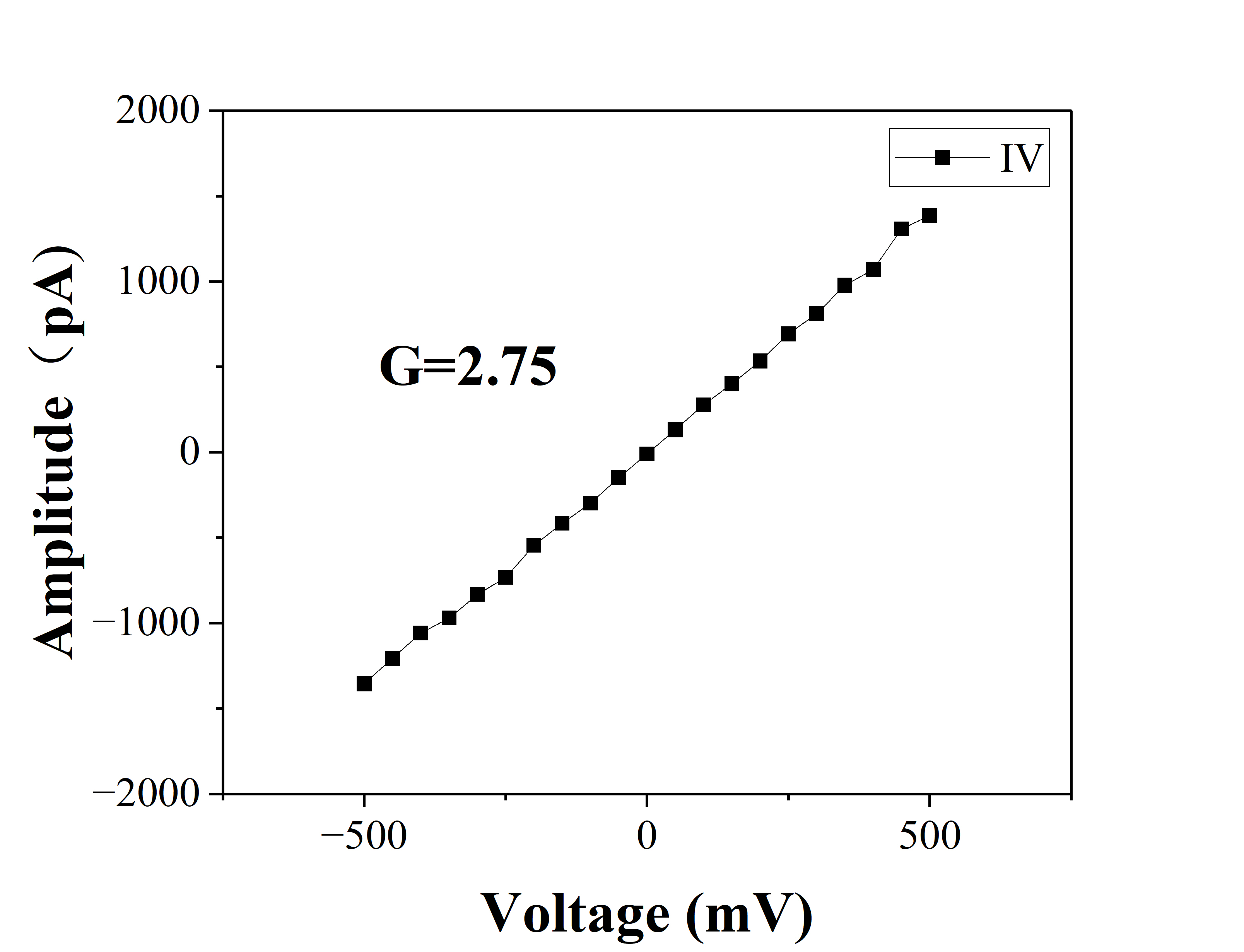


Fig S2 I-V curve of the nanopore in 1 M KCl,10 mM Tris, 1 mM EDTA, pH 8.0. The pore size was 3 nm.


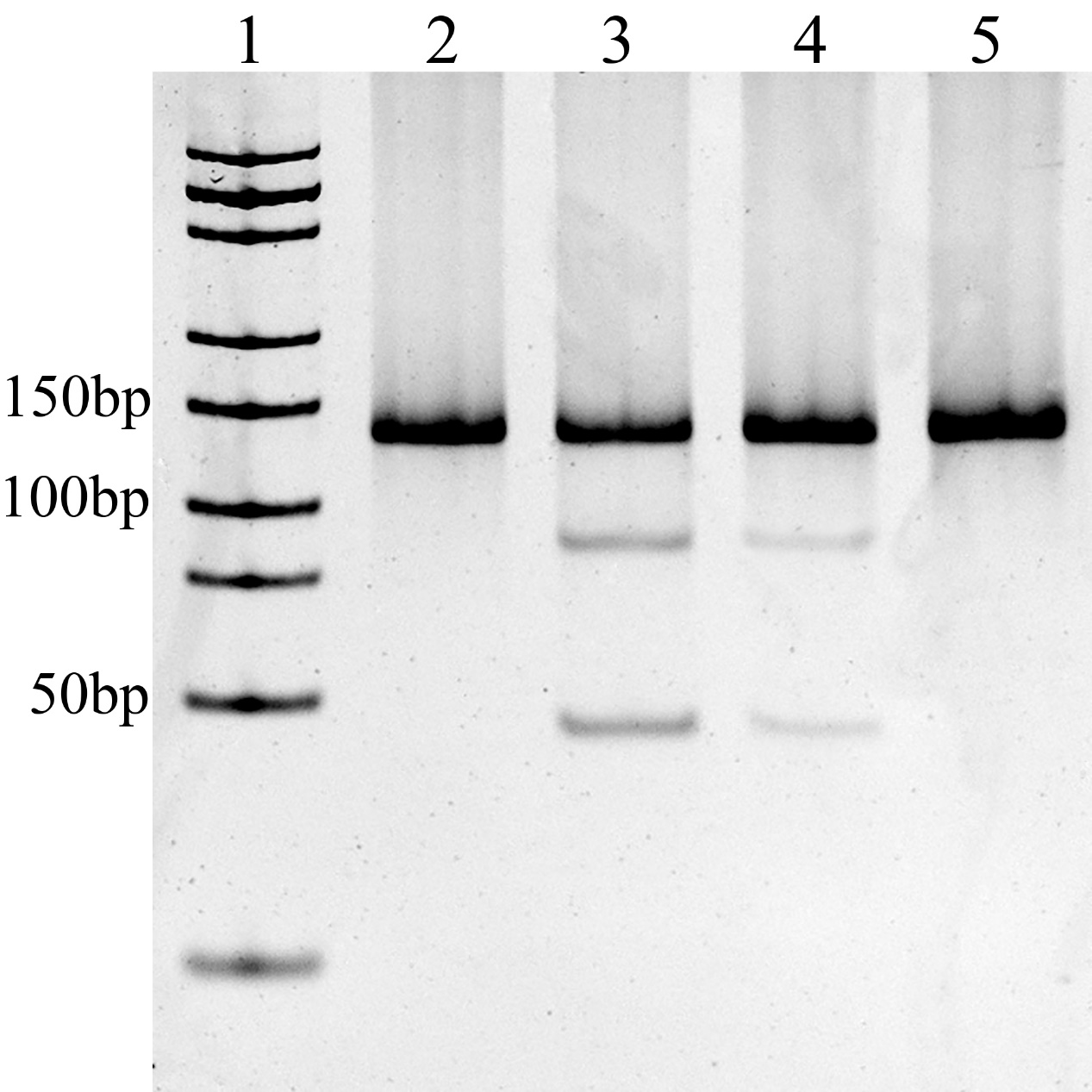
Fig S3 Optimal reaction temperature for T7E1 digestion. Lane 1: DNA marker (25-500bp), Lane 2: 25℃, Lane 3: 37℃, Lane 4: 45℃，Lane 4: 55℃.


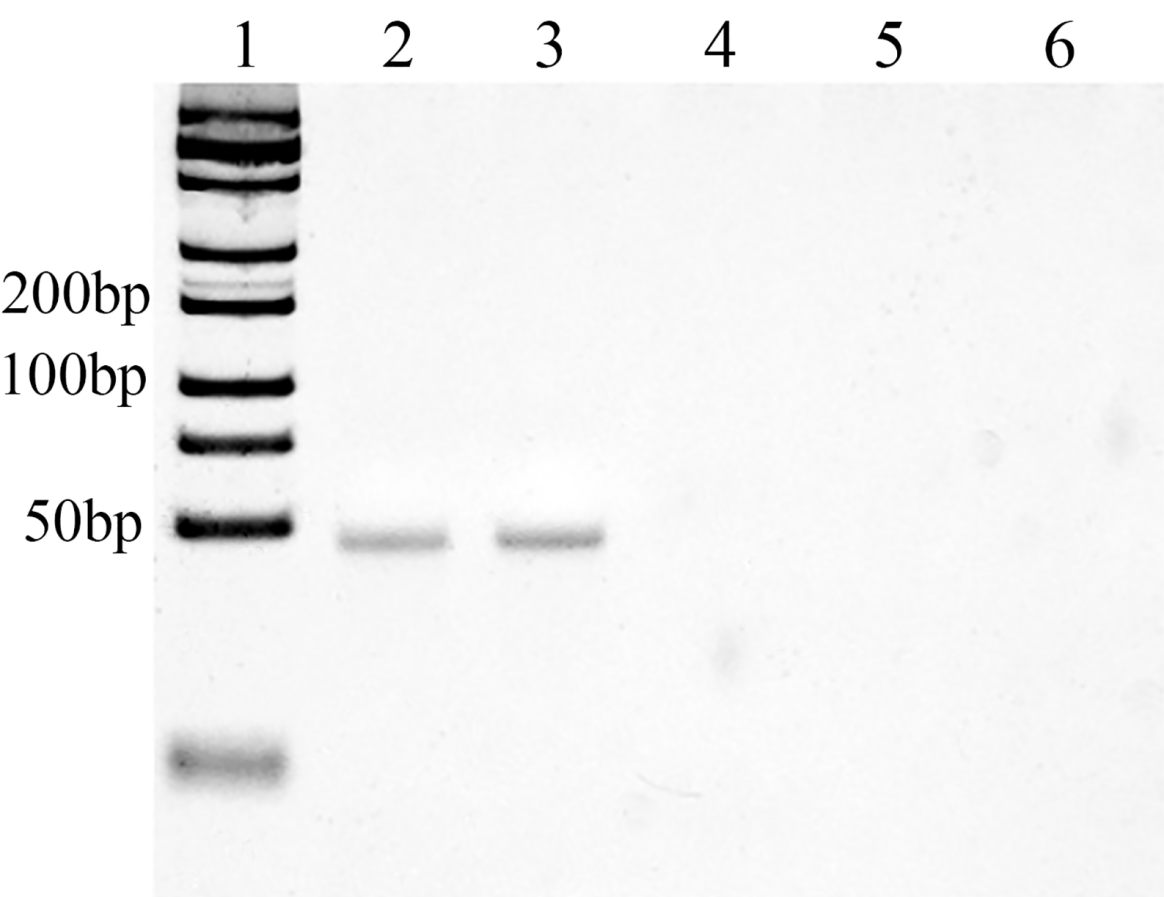


Fig S4 DNAs were captured by probes, and then digested by T7E1. The DNA fragments, generated after digestion, were finally purified using magnetic beads. Lane 1: DNA marker (25-500bp); Lane 2: 0.3μM EGFR/L858R heteroduplex; Lane 3: 0.3μM L858R, Lane 4: 0.3μM EGFR. Lane 5: 0.3μM K-RAS. Lane 6 0.3μM G12D.


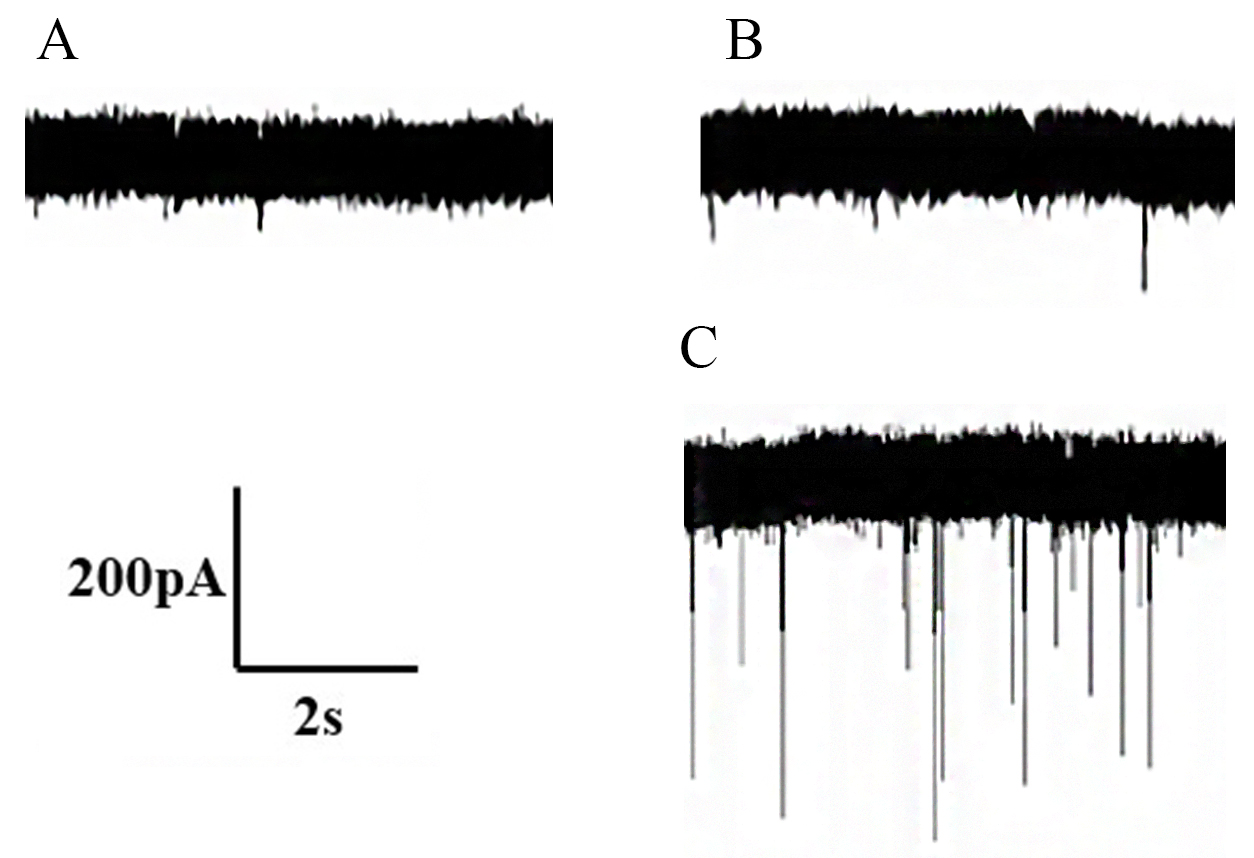


Fig S5 (A) Current signal in the presence of RAS (B) Current signal in the presence of G12D. (C) Current signal in the presence of L858R.


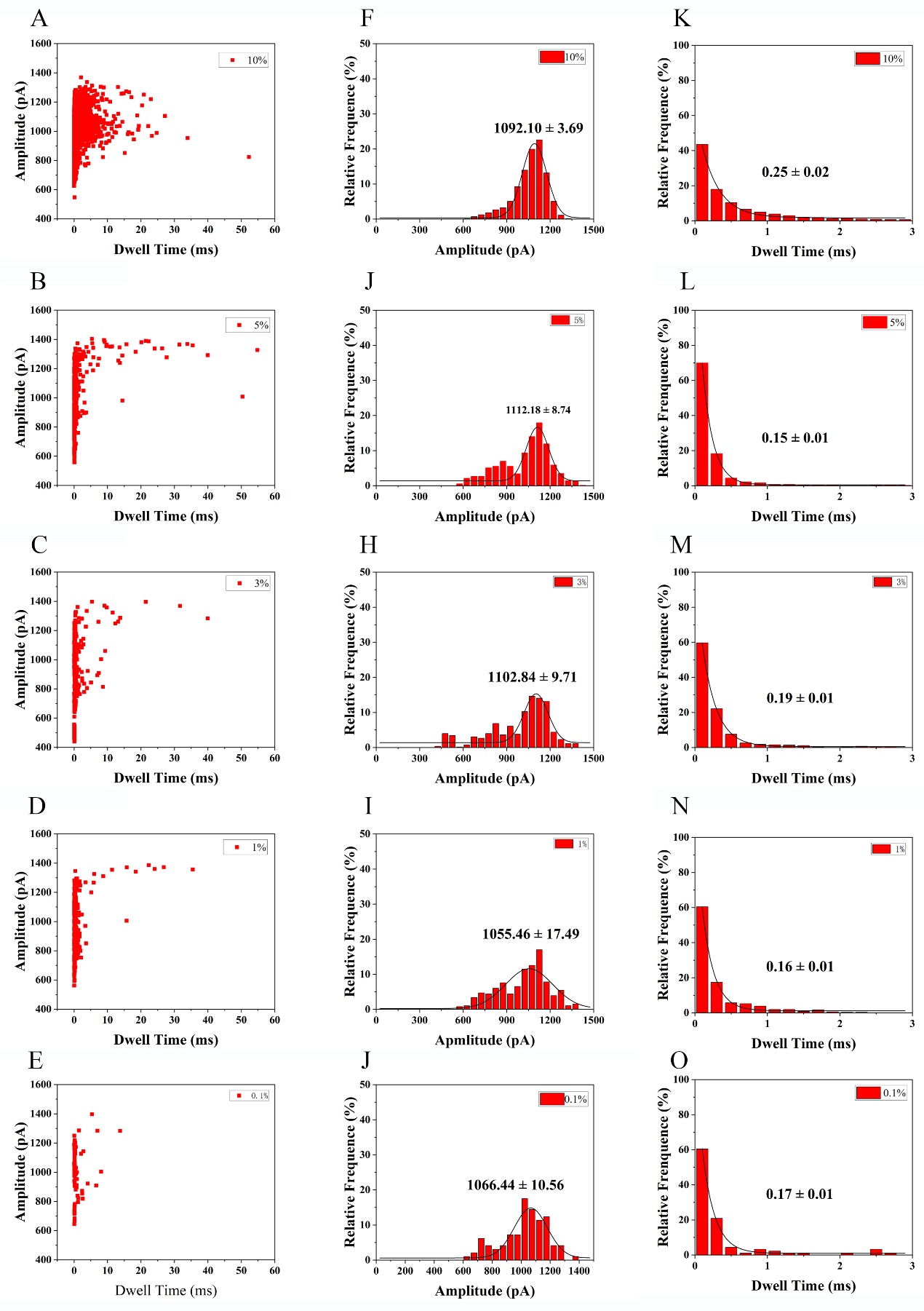


Fig S6 Detection of L858R at various concentration ratio. (A-E) Scatter plots of the amplitude of current blockade with dwell time; (F-J) Gaussian distributions of normalized histograms for current amplitude; (H-O) Normalized histograms of dwell time with fitting method of exponential decay.
